# Supplementary material for: Bioengineering of Soybean Oil and Its Impact on Agronomic Traits
Source: Int J Mol Sci. 2023 Jan 23;24(3):2256. doi: 10.3390/ijms24032256 (PMC9916542; doi:10.3390/ijms24032256)
Supplement: Supplementary file 1 [file ijms-24-02256-s001.zip › ijms-2150530-supplementary.pdf]

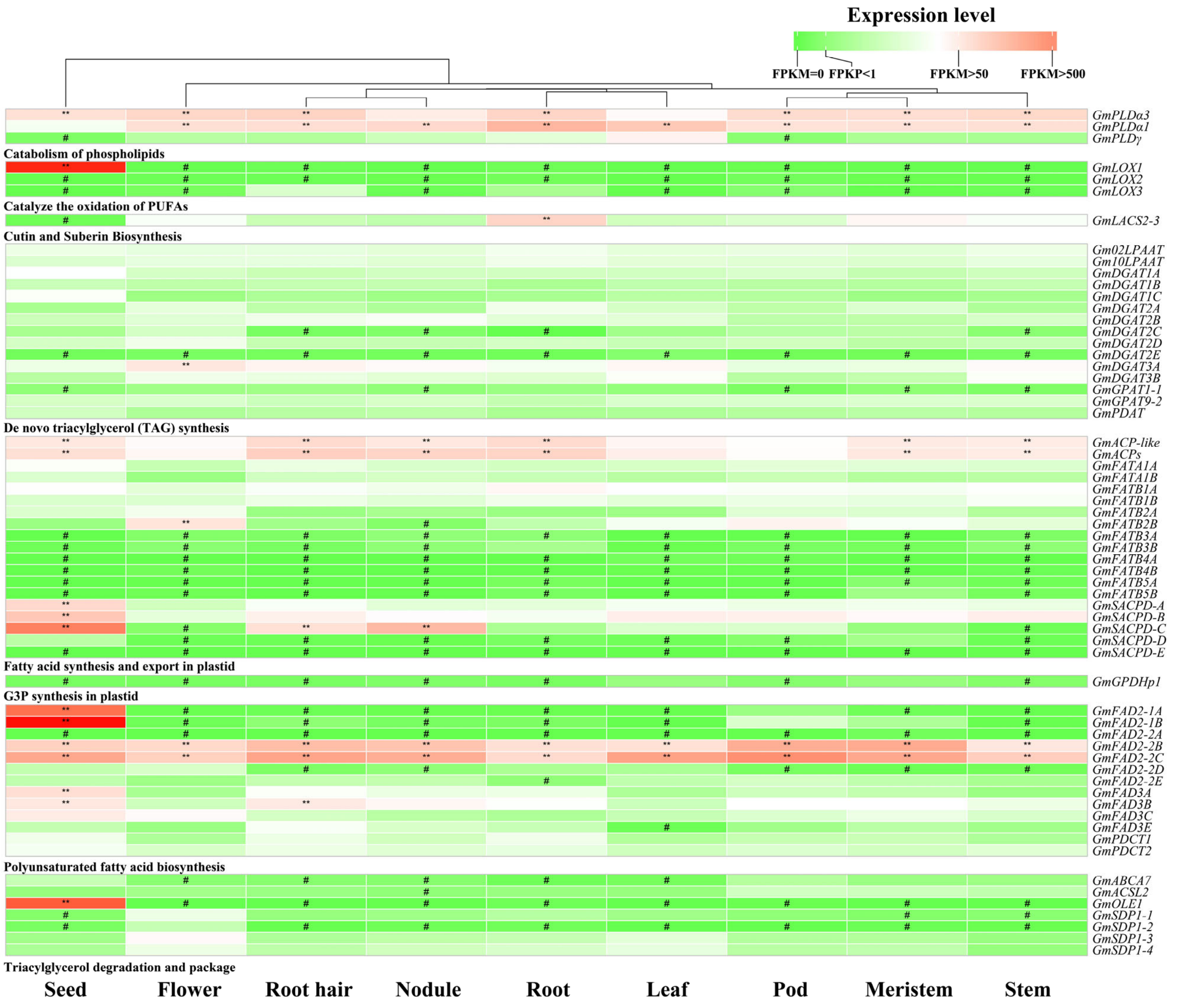

Figure S1. Expression levels of lipid related genes in soybean tissues. The heatmap showing tissue-specific expression profiles was generated using the log2-transformed (Fragments Per Kilobase of exon model per Million mapped fragments (FPKM) + 1) values of lipid related genes. High expression (FPKM > 50) is indicated with "**", while low expression (FPKM < 1) is indicated by "#". Brackets on the top show the results of column cluster analysis. The heatmap above was generated on the basis of RNA-seq data from the website (https://phytozome-next.jgi.doe.gov/).
